# A Spontaneous Mutation of the Rat *Themis* Gene Leads to Impaired Function of Regulatory T Cells Linked to Inflammatory Bowel Disease

**DOI:** 10.1371/journal.pgen.1002461

**Published:** 2012-01-19

**Authors:** Marianne Chabod, Christophe Pedros, Lucille Lamouroux, Céline Colacios, Isabelle Bernard, Dominique Lagrange, Daniela Balz-Hara, Jean-Francois Mosnier, Christian Laboisse, Nathalie Vergnolle, Olivier Andreoletti, Marie-Paule Roth, Roland Liblau, Gilbert J. Fournié, Abdelhadi Saoudi, Anne S. Dejean

**Affiliations:** 1 UMR Inserm, U1043, Toulouse, France; 2 UMR CNRS, U5282, Toulouse, France; 3 Université de Toulouse, UPS, Centre de Physiopathologie de Toulouse Purpan (CPTP), Toulouse, France; 4 Université de Nantes, Faculté de Médecine, EA Biométadys, Nantes, France; 5 UMR INRA ENVT 1225, Interactions Hôtes Agents Pathogènes, Ecole Nationale Vétérinaire de Toulouse, Toulouse, France; The Jackson Laboratory, United States of America

## Abstract

Spontaneous or chemically induced germline mutations, which lead to Mendelian phenotypes, are powerful tools to discover new genes and their functions. Here, we report an autosomal recessive mutation that occurred spontaneously in a Brown-Norway (BN) rat colony and was identified as causing marked T cell lymphopenia. This mutation was stabilized in a new rat strain, named BN^m^ for “BN mutated.” In BN^m^ rats, we found that the T cell lymphopenia originated in the thymus, was intrinsic to CD4 T lymphocytes, and was associated with the development of an inflammatory bowel disease. Furthermore, we demonstrate that the suppressive activity of both peripheral and thymic CD4^+^ CD25^bright^ regulatory T cells (Treg) is defective in BN^m^ rats. Complementation of mutant animals with BN Treg decreases disease incidence and severity, thus suggesting that the impaired Treg function is involved in the development of inflammatory bowel disease in BN^m^ rats. Moreover, the cytokine profile of effector CD4 T cells is skewed toward Th2 and Th17 phenotypes in BN^m^ rats. Linkage analysis and genetic dissection of the CD4 T cell lymphopenia in rats issued from BN^m^×DA crosses allowed the localization of the mutation on chromosome 1, within a 1.5 megabase interval. Gene expression and sequencing studies identified a frameshift mutation caused by a four-nucleotide insertion in the *Themis* gene, leading to its disruption. This result is the first to link Themis to the suppressive function of Treg and to suggest that, in Themis-deficient animals, defect of this function is involved in intestinal inflammation. Thus, this study highlights the importance of *Themis* as a new target gene that could participate in the pathogenesis of immune diseases characterized by chronic inflammation resulting from a defect in the Treg compartment.

## Author Summary

Deciphering the genetic basis of human diseases and understanding the function of mammalian genes are among the main challenges for today's geneticists. In this regard, rodent models represent invaluable tools to identify new genes and to study the mechanisms of action of genes implicated in human diseases. Here, we identified a spontaneous mutation responsible for a reduction of blood CD4 T lymphocyte counts in a rat strain. The mutant rats showed a high incidence of inflammatory bowel disease, which was associated with skewed cytokine secretion by effector CD4 T cells towards Th2 and Th17 and with impairment of the suppressive activity of the regulatory CD4 T cells (Treg). The contribution of Treg was further evidenced by experiments showing that transfer of Treg from normal BN rats to mutant animals prevented the occurrence of bowel lesions. By genetic mapping the lymphopenia, we identified a disruption of the *Themis* gene. This result is the first to link Themis to the suppressive function of Treg and to suggest that, in Themis-deficient animals, a defect of this function predisposes to intestinal inflammation. Thus, this new rat model highlights key roles of Themis both in regulating the immune system and in maintaining intestinal homeostasis.

## Introduction

Immune-mediated diseases are multifactorial disorders, resulting from complex interactions between multiple genes and the environment [1]. Deciphering the genetic bases of these diseases is a difficult task. Genetic association studies in human have recently identified numerous risk loci associated with susceptibility to immune-mediated diseases [2]–[4], but few causal genes have been unambiguously identified so far as being directly involved in a particular disease. In addition, identifying the exact mechanisms of action of these genes often remains a significant barrier to progress.

Mouse and rat models of human diseases are simplified systems in which environmental factors are under control and genetic heterogeneity is eliminated. They represent invaluable tools to identify new genes involved in human diseases. They also offer efficient systems for studying in-depth mechanisms of gene action. Two different and complementary approaches can be followed to investigate the function of a single gene and the pathological consequences of its dysfunction. First, the gene-driven approach can be used to investigate the consequences of the modification or invalidation of a known gene, using genetically modified animals such as knock-out and knock-in models. Second, the phenotype-driven approach aims at elucidating the gene and mechanisms involved in a given phenotype, identified either after a spontaneous mutation occurring in an animal colony [5], or generated by random mutagenesis with a mutagen such as N-ethyl-N-nitrosourea (ENU) [6].

Here, we report the identification of an autosomal recessive mutation responsible for T cell lymphopenia, which arose spontaneously in our Brown-Norway (BN) rat colony. This mutation was associated with a high incidence of inflammatory bowel disease (IBD), skewed cytokine profile of effector T cells towards Th2/Th17 and inefficient natural regulatory CD4 T lymphocytes (Treg). We thus sought to identify the mutated gene by positional cloning, and found a disruption of the *Themis* gene. Our study provides the first evidence for a role of Themis in CD4 T cell functions and in digestive tract homeostasis.

## Results

### BN^m^ rats exhibit a defect in thymic CD4 T cell development

**Figure 1:**
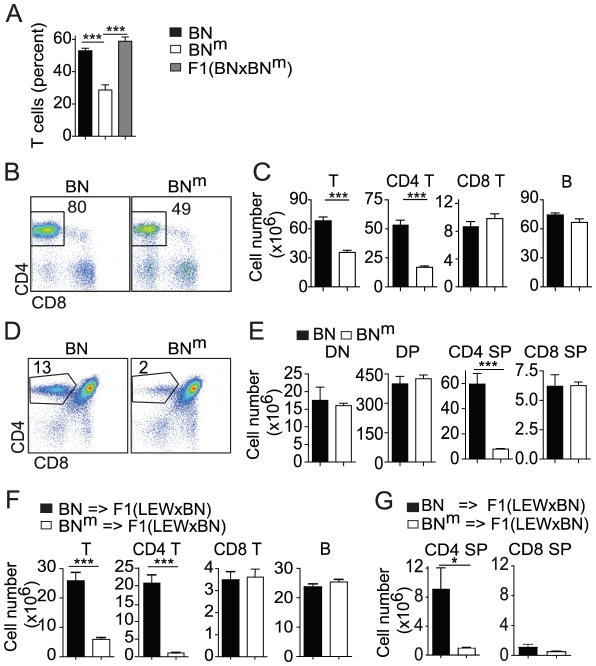
BN^m^ rats exhibit a T cell autonomous lymphopenia restricted to CD4 T cells. (A) Percentage of TCRαβ positive cells in PBMC from BN (n = 15, black bars), BN^m^ (n = 9, white bars) and (BN×BN^m^) F1 (n = 5, grey bars) rats. (B) CD4 and CD8 expression in BN and BN^m^ spleen T cells; numbers indicate the cell percentages in the outlined area. (C) Absolute numbers of BN (n = 4) and BNm (n = 9) T cells, CD4+ and CD8+ T cells, and B cells in spleen. Data in B and C are representative of five independent experiments. (D) CD4 and CD8 expression in BN and BN^m^ thymocytes; numbers indicate cell percentages in the outlined area. (E) Absolute numbers of BN (n = 5) and BN^m^ (n = 5) in thymocyte subsets (DN: double negative; DP: double positive; SP: simple positive). Data in D and E are representative of six independent experiments. (F) Absolute numbers of lymphocytes in spleen of lethally irradiated (LEW×BN) F1 rats reconstituted with BN (n = 6) or BN^m^ (n = 8) T cell-depleted bone marrow cells. Data are representative of three independent experiments. (G) Absolute numbers of donor CD4 SP and CD8 SP cells in thymus of sub-lethally irradiated (LEW×BN) F1 rats 15 days after intrathymic injection of DN thymocytes from BN (n = 4) and BN^m^ (n = 4) rats. (BN: black columns, BN^m^: white columns).

In the course of our ongoing studies on rat immunogenetics, we serendipitously observed that, within a litter of BN rats, ∼1/4 of the offspring showed a marked reduction of the proportion of T cells in the peripheral blood. Intercrosses rapidly led us to conclude that a spontaneous autosomal recessive mutation was responsible for this lymphopenia (Figure 1A). This mutation, which leads to an unambiguous and fully penetrant phenotype, was isolated and stabilized to homozygocity in a new rat strain named BN^m^, for “BN mutated”. Phenotypic analysis of cells from secondary lymphoid organs revealed that the proportion (Figure 1B) and absolute numbers (Figure 1C) of CD4 T cells were markedly reduced in spleen (Figure 1B–1C) and lymph nodes (LN) of BN^m^ rats (Figure S1). Numbers of CD8 T cells, B cells, macrophages, γδ T cells and NK cells were not significantly affected (Figure 1C and data not shown). In the thymus, while no decrease was observed in double negative (DN), double positive (DP), or CD8 single positive (SP) thymocytes, the proportion (Figure 1D) and absolute numbers (Figure 1E) of CD4 SP cells of BN^m^ rats were greatly reduced, showing that the CD4 T cell lymphopenia originates in the thymus. Results from bone marrow chimeras demonstrated that this CD4 T cell deficiency was intrinsic to hematopoietic cells and did not depend on the ability of thymic epithelial cells to support CD4 T cell differentiation (Figure 1F, Figure S2). Finally, by using intrathymic transfer of purified T cell precursors (DN thymocytes) from BN or BN^m^ rats into (LEW×BN) F1 rats, we documented that this defect was intrinsic to T cells rather than being dependent on the thymic environment (Figure 1G).

### BN^m^ rats develop inflammatory bowel disease

**Figure 2:**
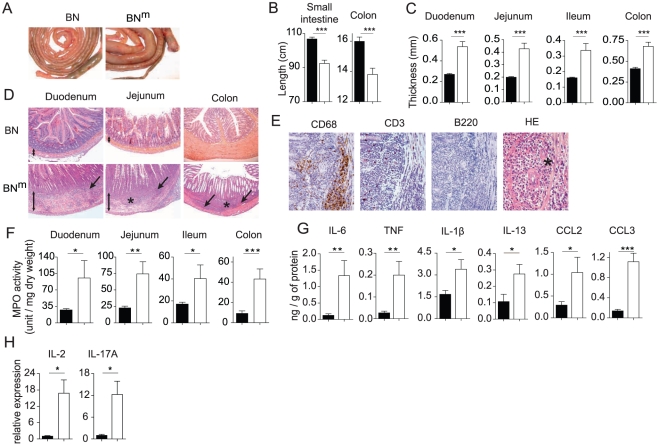
BN^m^ rats develop inflammatory bowel disease. (A) Small intestine and colon from 12 week-old BN and BN^m^ rats. (B, C) Length of small intestine and colon (B) and thickness of the duodenum, jejunum, ileum and colon (C) of BN (n = 10) and BN^m^ rats with macroscopic signs of intestinal lesions (n = 7). (D) Hematoxylin-eosin staining of duodenum, jejunum and colon from 8 to 10 week-old BN and BN^m^ rats. Images are representative of microscopic lesions observed in all affected BN^m^ rats (original magnification: 100 X). Stars indicate granulomas; arrows point to infiltration and double head arrows indicate the thickness of the intestinal wall. (E) CD68, CD3 and B220 immunoperoxydase staining on sections of jejunum from 8 week-old BN^m^ rats. Positive staining results in a brown reaction product. HE: hematoxylin-eosin staining showing the presence of polymorphonuclear leucocytes (original magnification: 400 X). (F) Myeloperoxidase (MPO) activity in the duodenum, jejunum, ileum and colon tissue samples from BN (n = 30) and BN^m^ rats with macroscopic lesions (n = 21). (G) Cytokine and chemokine protein expression in duodenum from BN (n = 7) and BN^m^ rats with macroscopic lesions (n = 7). (H) Relative mRNA expression of IL-2 and IL-17A in duodenum from BN (n = 4) and BN^m^ rats exhibiting intestinal macroscopic lesions (n = 7). (BN: black columns, BN^m^: white columns).

We looked for the presence of autoantibodies using sera from 12 week-old BN^m^ (n = 18) and BN rats (n = 5). We found that sera from BN^m^ rats contained autoantibodies that recognize gut (66%), kidney (50%), liver (50%) and lung (55%) tissues (Figure S3A). The antibodies recognized wall component(s) of arterioles and venules (liver, lung and kidney) and muscular layers in the intestine. In the kidney, a weak labeling was also observed in the glomerular mesangium. The nature of the labeled histological structures suggests that the autoantibodies are mainly raised against smooth muscles. These autoantibodies were undetectable in BN rats. We then examined organs from BN^m^ and age-matched control BN rats and showed the presence of tissue inflammation affecting the gut of BN^m^ rats. Indeed, macroscopic lesions were often observed in the intestinal tract from both male and female BN^m^ rats (Figure 2A). Shortening of the intestine (Figure 2B) and thickening of the intestinal wall (Figure 2C) were associated with inflammatory features including oedema, erythema, and increased mucus secretion. The earliest intestinal lesions were detected in 7-week-old animals, and by 12 weeks of age, disease prevalence reached 50 percent (Figure S3B). Histological analyses revealed slight to severe multifocal infiltration of the intestine wall, involving preferentially the muscular layers and the submucosa (Figure 2D, Figure S3C), whereas the intestinal mucosa did not display any infiltration or ulcerative lesions. Lesions were scattered all along the intestine (Figure 2D). The infiltration was predominantly composed of polymorphonuclear cells and macrophages (CD68^+^) with few T cells (CD3^+^) and no B cells (B220^+^) (Figure 2E). In addition, granulomas composed mainly of macrophages and polymorphonuclear leucocytes were often observed (Figure 2D–2E). The infiltration of polymorphonuclear cells was confirmed by a significant increase in myeloperoxidase (MPO) activity in the duodenum, jejunum, ileum and colon samples from BN^m^ rats (Figure 2F). These inflammatory features were associated with increased levels of IL-6, TNF, IL-1β, IL-13, CCL2 and CCL3 in the intestinal tissues (Figure 2G). Transcript levels for IL-2 and IL-17 were also highly increased in the inflamed tissues from BN^m^ rats (Figure 2H). To assess whether the CD4 T cell lymphopenia observed in BN^m^ rats is sufficient to explain IBD development, wild type BN rats were thymectomized and treated with anti-CD4 depleting mAb. Using this protocol, we were able to induce a CD4 lymphopenia similar to that observed in BN^m^ rats, in terms of number and phenotype of CD4 T cells (data not shown). However, lymphopenic BN rats did not develop clinical or histological manifestation of IBD (data not shown). These results suggest that the mutated gene is responsible for IBD occurrence by other mechanisms than the simple induction of CD4 lymphopenia. Thus, BN^m^ rats harbor a mutation that predisposes to inflammatory lesions of the whole intestine that share pathological features with human IBD.

### BN^m^ rats exhibit spontaneous CD4 T cell activation and skewed cytokine production

**Figure 3:**
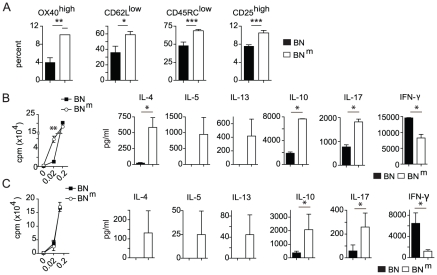
BN^m^ rats exhibit spontaneous T cell activation and skewed cytokine production. (A) Proportions of OX40^high^, CD62L^low^, CD45RC^low^ and CD25^high^ among CD4 T cells from spleens of BN (n = 4) and BN^m^ (n = 8) rats. (B) Proliferation was assessed by [3H] Thymidine uptake in total CD4 T cells isolated from BN (n = 4) and BN^m^ (n = 4) spleens after stimulation for 48 hours with anti-TCR mAb and increasing concentrations of anti-CD28 mAb (left panel). Cytokine production by CD4 T cells stimulated with anti-TCR mAb and 0.2 µg/ml of anti-CD28 mAb for 48 hours (right panels). (C) Proliferation (left panel) and cytokine production (right panels) by naive CD62L+ CD4 T cells isolated from BN (n = 4) and BN^m^ (n = 4) spleens and stimulated as described above. Data are representative of three independent experiments. (BN: black columns, BN^m^: white columns).

As abnormal T cell activation is often associated with inflammatory diseases, we assessed the phenotype and functions of peripheral T cells in BN^m^ rats. We observed an increased proportion of activated and memory-like CD4 T cells (OX40^high^ CD62L^low^ CD45RC^low^ CD25^high^) in spleen (Figure 3A) and mesenteric lymph nodes (mLN) (Figure S4A). Moreover, CD4 T cells purified from BN^m^ spleen (Figure 3B) and mLN (Figure S4B) exhibited an increased capacity to proliferate under low co-stimulatory conditions. At high doses of stimulation, CD4 T cells from BN and BN^m^ rats proliferated equally well, but BN^m^ CD4 T cells produced higher amounts of IL-4, IL-5, IL-13, IL-10, IL-17 and lower amounts of IFN-γ (Figure 3B and Figure S4B). To assess whether these phenotypes were the consequence of the higher proportion of activated CD4 T cells in BN^m^ rats, similar experiments were performed with purified naive CD62L^+^ CD4 T cells. Under the same conditions of stimulation, although naive BN and BN^m^ CD4 T cells proliferated equally well, they still differed in their cytokine production, particularly for IL-10, IL-17 and IFN-γ (Figure 3C). Finally, we analyzed the contribution of CD4 T cells to IBD development in BN^m^ rats and found that their depletion reduced both IBD incidence and severity (Figure S4C), indicating that CD4 T cells are involved in IBD development. Collectively, these data demonstrate that the mutation present in BN^m^ rats influences CD4 T cell activation and differentiation into Th1/Th2/Th17 subsets and that CD4 T cells contribute to IBD development.

### The suppressive activity of CD4^+^ CD25^bright^ regulatory T cells from BN^m^ rats is defective

**Figure 4:**
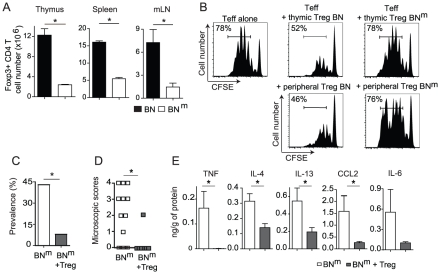
Impaired suppressive function of BN^m^ Treg is involved in the development of intestinal lesions. (A) Absolute numbers of Foxp3+ CD4+ T cells in thymus, spleen and mLN from BN (n = 7) and BN^m^ (n = 13) rats. Data are representative of three independent experiments. (B) Suppressive activity of thymic CD25^bright^ CD4+ SP cells (top panel) and peripheral CD25^bright^ CD4+ T cells (bottom panel) from BN or BN^m^ rats was assessed in co-culture experiments with CFSE-labeled naive LEW CD4 T cells as effector cells. Proliferation was assessed by CFSE dilution (percentages indicate the proportion of CFSE^low^ cells). Data are representative of three independent experiments. (C, D) Disease frequency (C) and duodenum microscopic scores (D) in 12 week-old BN^m^ rats injected with PBS (white column or symbols; n = 21) or with 4.10^6^ BN CD25^bright^ CD4+ T cells (grey columns or symbols; n = 12) at 4 weeks of age. (E) Cytokine protein expression in duodenum from control BN^m^ rats with microscopic intestinal scores (white columns; n = 8) and from BN^m^ rats transferred with CD25^bright^ CD4+ T cells (grey columns; n = 12).

Immune-mediated inflammation of the gastrointestinal tract is commonly observed when natural Treg, defined by the expression of Foxp3 and CD25, are dysregulated or deficient [7]. We thus wondered whether the IBD developing spontaneously by BN^m^ rats was the consequence of a quantitative and/or qualitative defect in the Treg compartment. The absolute number of Treg was indeed reduced in the thymus, spleen and LNs from BN^m^ compared to BN rats (Figure 4A). The proportion of CD4 Treg in BN^m^ thymus was decreased, indicating that the mutated gene is also involved in thymic Foxp3^+^ CD4 T cell development (Figure S5A). In contrast, this proportion was increased in spleen and mLNs (Figure S5A), suggesting that this quantitative defect of Treg is not responsible for IBD development. We therefore assessed the suppressive activity of Treg in co-culture experiments, using either thymic or peripheral CD25^bright^ CD4^+^ T cells (more than 85% being Foxp3^+^). As shown in Figure 4B, peripheral as well as thymic Treg from BN^m^ rats showed almost no suppressive activity, while Treg from BN rats exerted a potent inhibition of the proliferation of CFSE-labeled effector CD4^+^ T cells. Similar results were obtained using thymidine incorporation as a read-out of T cell proliferation (Figure S5B). Collectively, these data indicate that the mutation carried by BN^m^ rats plays a crucial role in the development and function of Foxp3^+^ Treg.

To investigate the relationship between this defect in Treg and the development of IBD, we assessed whether Treg with normal suppressive functions could prevent the development of inflammatory bowel lesions in BN^m^ rats. To this end, we performed adoptive transfer experiments of Treg from BN rats into BN^m^ rats. We found that transfer of 4.10^6^ CD25^bright^ CD4^+^ T cells from BN rats into 4 week-old BN^m^ rats significantly protected the recipients from intestinal inflammation (Figure 4C–4D). Indeed, while the prevalence of intestinal lesions in 12 week-old BN^m^ rats was 43% (9/21), only 8% (1/11) BN^m^ rats transferred with CD25^bright^ CD4^+^ T cells from BN rats showed macroscopic lesions (Figure 4C). Histological analyses confirmed this observation, showing lower scores of lesions in BN^m^ rats transferred with BN Treg, with a decreased cellular infiltration of the intestine wall and the absence of granuloma (Figure 4D). This prevention was associated with decreased amounts of TNF, IL-4, IL-13, IL-6 and CCL2 in intestinal tissues (Figure 4E). Altogether, these results led us to conclude that in BN^m^ rats, the defect in Treg suppressive function contributes to the development of IBD.

### *Themis* is disrupted in BN^m^ rats

**Figure 5:**
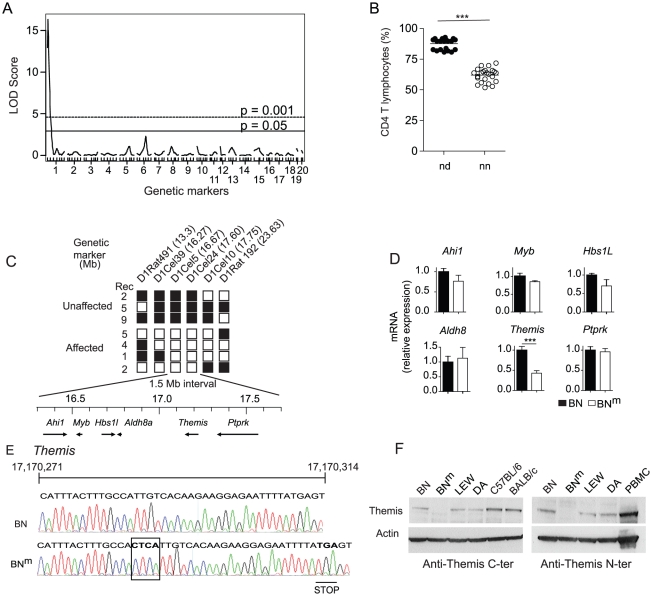
BN^m^ rats carry a disrupted *Themis* gene. (A) Genome scan for loci controlling the percentage of CD4 T cells in the blood of 44 (BN^m^×DA)×BN^m^ rats. Horizontal lines represent genome-wide significance thresholds of 5% (significant) and 0.1% (highly significant) as determined by permutation tests. (B) Percentages of CD4 T cells in 44 (BN^m^×DA)×BN^m^ backcross rats classified according to their genotypes at the microsatellite marker of chromosome 1 nearest to the QTL. In each group, horizontal bars represent the mean values. nn: homozygous BN (n = 23); nd: heterozygous BN-DA (n = 21). (C) Fine mapping of the BN^m^ mutation in 28 (BN^m^×DA) F2 or (BN^m^×DA)×BN^m^ backcross rats, among which 16 showed normal proportions of CD4 T lymphocytes (Unaffected) and 12 showed CD4 T cell lymphopenia (Affected). The position of each microsatellite marker on chromosome 1 is indicated in megabases (Mb). White: homozygous BN^m^; black: heterozygous BN^m^/DA. Rec: number of rats characterized by a given recombination. The physical map of the critical 1.5 Mb interval containing 6 genes is shown underneath. (D) Relative mRNA expression of the 6 genes in BN^m^ (n = 4) and BN (n = 4) thymocytes. Data are representative of two independent experiments. (E) Electrophoregram, nucleic acid sequences and corresponding amino acids of the BN and BN^m^ Themis gene in the region surrounding the 4 nucleotide insertion corresponding to the BN^m^ mutation (boxed). (F) Immunoblot analysis of Themis and β-actin in thymocytes from BN, BN^m^, LEW and DA rats, C57BL/6 mice and in human PBMC using anti-Themis antibodies specific either for the mouse C-terminal (left panel) or the human N-terminal (right panel) portion of the protein.

To localize the gene responsible for CD4 T cell lymphopenia, 44 rats obtained from a (BN^m^×DA)×BN^m^ backcross were genotyped using 98 microsatellite markers polymorphic between the parental strains and distributed across the genome. A genome-wide scan was performed using R/qtl software and permutations of the phenotypes to define genomewide LOD thresholds of 5% and 0.1% (termed here as significant and highly significant, respectively). As shown on Figure 5A, a highly significant quantitative trait locus (QTL) controlling the percentage of CD4 T cells was found in a 8 cM interval of chromosome 1 (peak LOD score of 16.45; p<0.0001). This QTL accounts for 82% of the total variance of the trait. As shown on Figure 5B, when the 44 rats are separated according to their genotype at the microsatellite marker nearest to the QTL peak, D1Rat1, rats with the ‘nd’ genotype have, similarly to their F1 parents, high CD4 T cell percentages. In contrast, rats with the ‘nn’ genotype have low percentages, in the range of those observed in their inbred backcross parents BN^m^. The trait distribution in the 44 rats is clearly discontinuous and falls into two equally populated classes that are separated by the genotype at the D1Rat1 marker. The percentage of CD4 T cells is therefore controlled by a mutant allele at a single locus in the 8 cM QTL region of chromosome 1. Further genotyping using 7 additional microsatellite markers allowed the localization of the gene on chromosome 1 within a ∼10.3 Mb interval. To reduce this region further, 12 additional markers were used to genotype 379 rats obtained from (BN^m^×DA)×BN^m^ or (BN^m^×DA)F2 crosses. As shown on Figure 5C, only the genotypes at markers mapping to a small 1.5 Mb interval of the initial region between 16.27 Mb (D1Cel39) and 17.75 Mb (D1Cel10) were able to separate rats with CD4 T cell lymphopenia and rats with normal CD4 counts. According to databases RGD (Rat Genome Database: http://rgd.mcw.edu/) and Ensembl (http://www.ensembl.org/Rattus_norvegicus/Info/), this region contains six genes (Figure 5C), including *Themis* and *Ptprk*, which have both been described as involved in T cell development [8]–[12]. Study of mRNA expression of these six genes in thymocytes from BN^m^ rats by quantitative RT–PCR revealed a significant decrease only for *Themis* mRNA expression (Figure 5D). Sequencing of coding sequences revealed a frameshift mutation in the *Themis* gene of BN^m^ rats, resulting from a four-nucleotide insertion, which introduces a premature stop codon (Figure 5E). By contrast, no polymorphism was found between the BN and BN^m^ strains in the sequences coding for *Ptprk* (data not shown). Consistent with these findings, western blot analysis using Themis-specific antibodies that recognize either the N- or C-terminal fragments of the protein revealed no detectable Themis expression in BN^m^ thymocytes, whereas it was detectable at the predicted size of 73 kDa in protein extracts from BN rats (Figure 5F). Together, these data strongly suggest that the CD4 T cell lymphopenia observed in BN^m^ rats results from a disruption of the *Themis* gene and that the BN^m^ rat thus represents a spontaneous Themis knock-out rat model.

## Discussion

In the present study, we serendipitously discovered a spontaneous mutation in the BN rat strain, which leads to lymphopenia and increased susceptibility to inflammatory bowel disease. We have mapped this mutation to an insertion of four nucleotides causing the disruption of the gene encoding the Themis protein. Themis (thymocyte-expressed molecule involved in selection), also known as Gasp (Grb2-associating protein), has been recently identified in mice as an essential component of the early TCR signaling machinery [13] that plays an important role in positive selection during thymic T cell development [8]–[11]. Indeed, Themis-deficient mice have considerably fewer thymic and peripheral CD4 and CD8 T cells, although the CD4 population is affected more severely than CD8 population [8]–[11]. In our rat model, the Themis deficiency was found to affect more specifically the CD4 T cell compartment. In the effector T cell compartment, the absence of Themis was associated with CD4 T cell activation, increased production of Th17-Th2 cytokines and reduction of IFN-γ production. Concerning the Treg compartment, not only was the proportion and absolute numbers of thymic CD4 Treg decreased in Themis-deficient rats, but the Themis-deficient CD4^+^ CD25^bright^ T cells also exhibited a defect in their suppressive activity. We found that this defect contributes to the spontaneous development of IBD in BN^m^ rats since their complementation with BN Treg decreases disease incidence and severity. Finally, the majority of diseased BN^m^ rats (77%) harbors autoantibodies against intestinal tissues. Thus, our study highlights the contribution of Themis to both the immune system and intestinal homeostasis.

BN^m^ rats develop an inflammatory disease that affects preferentially the gut tissue. This might be related to the BN genetic background, since we found that BN rats were highly sensitive to TNBS induced colitis (our unpublished data). Of note, no pathological manifestations have been reported in Themis-deficient mice. Differences in species, or the particular BN genetic background might explain this distinct susceptibility to IBD. This raises interesting questions regarding the basis for the similarities and differences between the roles of Themis in regulating the immune system and the digestive tract homeostasis in mice versus rats. Among the rodent models of T cell immunodeficiency for which the development of an IBD has been reported, the Long-Evans Cinnamon (LEC) rat model is of particular interest. Similarly to BN^m^ rats, an arrest in the maturation of thymocytes from CD4^+^CD8^+^ to CD4^+^ SP was observed in LEC rats [14]. While two groups initially attributed this defect to the invalidation of the *Ptprk* gene [12], [15], a subsequent report suggested that, in LEC rats, immunodeficiency results from a combined deletion of both *Ptprk* and *Themis* genes [16]. LEC rats also develop IBD, but the disease is different from the IBD observed in BN^m^ rats. Indeed, inflammatory lesions in LEC rats are restricted to the cecum and colon, while inflammation affects the whole intestine in BN^m^ rats. Moreover, the disease in LEC rats is associated with an increased secretion of Th1 cytokines while it is associated with a Th2/Th17 cytokine imbalance in BN^m^ rats. Finally, LEC rats, but not BN^m^ rats, also develop Wilson's disease. These differences between LEC and BN^m^ rats can be attributed to differences in genetic backgrounds, or more likely, to a complex interplay between Ptprk and Themis in LEC rats. The present study clearly establishes that *Themis* impacts on Treg suppressive function independently of *Ptprk* and that this effect is involved in the development of IBD lesions.

Our results document that Themis acts as a key player in the suppressive function of Treg at an early stage of thymic development, independently of lymphopenia and inflammation. Indeed, the defect in Treg in BN^m^ rats is present in CD4 SP thymocytes and is observed in the periphery of 6 week-old animals, at a time when inflammatory bowel lesions have not yet developed. The impaired function of Treg observed in BN^m^ rats probably contributes to the physiopathology of IBD since complementation of BN^m^ rat with wild type Treg inhibited disease development. Finally, analysis of the expression of CTLA-4, GITR, IL10-R, TGF-βR and Foxp3 by quantitative RT-PCR did not reveal any differences between Treg from BN and BN^m^ rats (data not shown), indicating that defective expression of these molecules is not the cause of impaired Treg function. The impact of Themis on T cell thymic selection, and on mature T cell functions likely results from its role in proximal signaling downstream of the TCR. Indeed, after TCR triggering, Themis is phosphorylated on tyrosine residues within seconds, both in mouse and human T cells [13]. This phosphorylation depends on LAT, Lck and SLP-76 [13] and allows Themis to act as an adaptor protein that binds to LAT, Grb2 and PLC-γ. Consistent with our data, mice deficient for Themis partners such as PLC-γ or LAT also develop immune-mediated diseases linked to defects in the suppressive functions of CD4 Treg [17]–[19]. Moreover, despite normal Foxp3 expression, Themis, LAT or PLC-γ deficient CD4^+^ CD25^+^ T cells are ineffective in controlling the proliferation of effector T cells [17], [18], [20]. Together, these data suggest that the Themis/LAT/PLC-γ signaling hub is mandatory for the suppressive functions of Foxp3^+^ CD4 T cells.

In humans, the etiology of IBD is still unknown. The consensus hypothesis is that, in genetically predisposed individuals, both environmental factors such as the luminal content and endogenous factors (defects in barrier, vascular or neuronal functions) trigger an uncontrolled inflammatory response [21]. The main forms of IBD are Crohn's disease and ulcerative colitis, which differ mostly by the region of the gut where inflammation progresses and the depth of inflammatory damage. While ulcerative colitis progresses from the rectum towards the colon, presenting only superficial (mucosal) damage, Crohn's disease can affect all parts of the digestive tract and is characterized by transmural damage and inflammatory cell infiltration [22]. Lesions observed in Themis-deficient BN rats display several characteristics shared with Crohn's disease. Inflammation in BN^m^ rats involves the entire intestinal tract (from the duodenum to the distal colon) and appears patchy all along the digestive tract. Inflammatory cells infiltrates are found in deep layers of the intestine in BN^m^ rats (submucosa and muscle layers) and the granulomas are very similar to those seen in Crohn's disease [23], [24]. In contrast to Crohn's disease, however, IBD in Themis-deficient rats does not show superficial mucosa damage, diarrhea or bleeding.

To date, genome-wide association studies performed in patients suffering from IBD [25], [26] have not identified the genomic region bearing Themis as a susceptibility locus. Such an association, however, has been found in patients suffering from celiac disease, another multi-factorial immune-mediated disease of the intestinal tract [27]. Both diseases share common physiopathological features, such as an initial increase in intestinal permeability and the T helper pattern of the immune response [28]. Accordingly, the two diseases can occur concomitantly in families or individual patients and the prevalence of IBD is increased in patients suffering from celiac disease [29], [30]. Recently, a meta-analysis of genome-wide association data from celiac disease and Crohn's disease has identified several risk loci shared by both diseases [31], thus adding further support for common pathophysiological pathways and to the possible involvement of *THEMIS* in human IBD. Nowadays, it is estimated that the identified genes associated with IBD represent only a fraction of the genetic risk [2]. Many susceptibility genes still remain to be discovered. Gene identification in humans is hampered by several factors including variable penetrance, low relative risk associated to a single disease allele, epistasis, genetic heterogeneity of human populations and variability of environmental factors [32]. Thus, animal models of IBD, which develop under stable and controlled laboratory conditions, represent a very useful tool to study the pathogenesis of IBD by identifying the genes involved and deciphering their mechanisms of action. We propose that *Themis*, due to its role on CD4 T cell functions and immune system homeostasis, should now be considered as a candidate gene for IBD susceptibility, as well as for other immune mediated diseases where a dysfunction of Treg is documented or suspected. The rat model we describe will probably help to understand how Themis directs Treg function, which is indeed an important issue given the pivotal role of Treg in maintaining immunological tolerance.

In conclusion, our study highlights the importance of spontaneous mutations in developing new animal models of human genetic disorders, which are invaluable tools for hunting new genes and analyzing their functions. We describe a rat model of spontaneous IBD that shares many features with Crohn's disease. We found a disruption of the gene encoding Themis, which leads to impaired regulatory T cell function, thus favoring IBD development. While the role of Themis in thymic selection has been previously established, our study provides the first evidence for a role of Themis in homeostasis of the digestive tract, through an effect on the suppressive function of natural CD4 Treg. We thus believe that Themis dysfunction could also participate in the pathogenesis of other diseases characterized by chronic inflammation resulting from a defect in the Treg compartment.

## Materials and Methods

### Animals

Brown-Norway (BN), Lewis (LEW) and Dark-Agouti (DA) rats were obtained from Janvier Laboratories (Le Genest-Saint-Isle, France) and bred and maintained under specific pathogen–free conditions in our facility, in accordance with European guidelines. The mutation responsible for CD4 T cell lymphopenia arose spontaneously in our colony of BN rats after brother and sister mating. Lymphopenic rats were intercrossed to create the new strain, homozygous for the recessive mutation, called BN^m^. This study was carried out in accordance with the recommendations in the Guide for the Care and Use of Laboratory Animals of the National Institutes of Health. The protocol was approved by the Committee on the Ethics of Animal Experiments (Licence number: 31259).

### Antibodies, flow cytometry, and isolation of leukocyte subpopulations

Cell suspensions were prepared from thymus, spleen, mLN or BM. The mAbs used were as follows: OX6 (anti-rat MCH class II), OX8 (anti-rat CD8α), OX27 (anti-rat RT1-A^c,n^), OX33 (anti-rat CD45RA), OX39 (anti-rat CD25), B5 (anti-rat RT1-A^a,b,l^), V65 (anti-rat TCRγδ), 3.2.3 (anti-rat NKR-P1), ED1 (anti-rat CD68), W3/25 (anti-rat CD4), R73 (anti-rat TCRαβ) and 341 (anti-rat CD8β). The mAbs used for flow cytometry were either prepared in our own laboratory or purchased from Biolegend, Abd Serotech, BD Biosciences and eBioscience. For isolation of CD4 T cells, cell suspensions prepared from spleen and LN were incubated with a mixture of the following mAbs: OX6, OX33, 3.2.3, V65 and OX8 and were negatively selected using anti-mouse IgG magnetic microbeads (Dynal, Oslo, Norway). For Treg purification, negatively selected CD4 T cells were stained with 341-FITC (anti-rat CD8β), R73-APC (anti-rat TCRαβ), W3/25-PB (anti-rat CD4), and OX39-PE (anti-rat CD25) and electronically sorted using a Facs Aria II-Sorp (BD Biosciences). The purity of the sorted cells was checked and was greater than 98%.

### Radiation bone marrow chimeras

(LEW×BN) F1 recipient male rats were subjected to 8.5 Gy total body irradiation (^137^Cs source) 1 day before transplantation with bone marrow from BN or BN^m^ rats. Recipients were sacrificed 16 weeks after transfer. The extent of hematopoietic cell replacement by donor cells was analyzed using RT1-A haplotype-specific mAbs and was consistently of at least 96%.

### Intrathymic transfer of DN thymocytes

(LEW×BN) F1 recipient male rats were subjected to 2.5 Gy total body irradiation (^137^Cs source) 1 day before intrathymic DN cell transplantation. DN cells were purified from the thymus by negative selection using anti-CD4 (W3/25) and CD8 (OX8) mAbs and anti-mouse IgG magnetic microbeads (Dynal, Oslo, Norway). Recipients were killed two weeks after transfer and thymus was analyzed for the origin of immune cells, using RT1-A haplotype-specific mAbs.

### Linkage analysis and fine mapping

For the initial genome screen, the percentage of CD4 T cells was assessed in 44 rats obtained from a (BN^m^×DA)×BN^m^ backcross. These rats were genotyped for 98 polymorphic microsatellite markers spread across the genome at 20–30 cM intervals, as already described [33]. The R/qtl software [34] was used for mapping loci controlling the phenotypic trait (QTLs) and genome-wide thresholds of 5% and 0.1% significance were derived from 10,000 permutations of shuffled phenotypes. Further genotyping using 7 additional microsatellite markers allowed the localization of the gene on chromosome 1 within a ∼10.3 Mb interval. Then, genetic dissection of the region harboring the mutated gene was conducted in (BN^m^×DA)×BN^m^ backcrosses and (BN^m^×DA) F2 hybrid rats. For this purpose, a genetic map with a high density of markers was constructed. Among the 379 rats that were genotyped and phenotyped for CD4 lymphopenia, 28 rats showed recombination within the ∼10.3 Mb and were further genotyped using 12 additional microsatellite markers, allowing the localization of the mutated gene within a ∼1.5 Mb interval. The sequences of the forward and reverse primers of the new markers used for the fine mapping of the genetic defect and designed *in silico* are the following:

D1Cel39: F-CCACAGTGTTTGAGTGAAAGGCCT;

  R-CATGATTGCTGTGCTGTGTGTGTG;

D1Cel5: F-CAAATTAGATACAATCCACTTGGG;

  R-TTCAGTAACCTGTATGAATGCTTA;

D1Cel24: F-TGCTCACGAGTCTATCTCCCAGGT;

  R-GGGCAGCTCTTGAGATTCAGGTG;

D1Cel10: F-AGCACCTGCATTCACAATGACACA;

  R-GCTTTTCAGCACTAGCCTTCCCTT

The physical map of the interval was extracted from the rat genome V3.4 assembly at the rat genome database (http://rgd.mcw.edu/).

### Quantification of mRNAs by RT–PCR

Total RNA was prepared using the RNeasy kit (Qiagen). cDNA generated by SuperScript III (Invitrogen) were analyzed using primers for the indicated genes. Real-time PCR was performed using SYBR green. Results were normalized to Atp5b expression levels. The primers used were as follows:

IL-2: F-AAGCAGGCCACAGAATTGAAAC;

  R-GCTGCAGAGCTCCGAGTTCAT

IL-17: F-AGCCGCAATGAGGACCCT;

  R-ATGTGGTGGTCCAACTTCCC

Ahi1: F-AGAGCGAGCCCATTCTTCTT;

  R-CTGTAGCGCTTCAACATTTCA

Aldh8: F-GATAGCCAAGCCCAGCGA;

  R-CCTGGTGGCACACCTGCT

Hbs1l: F-GAGAGCATGGCCTTTTGGTC;

  R-GTTGCCAATTTACCTGGTCCA

Myb: F-CCCCAAATATTCTTACGAGCTCTG;

  R-GTACGGTAAAGGCTTTGAGAACG

Ptprk: F-AGAAGCCTGCTTATGTGGAGA;

  R-TCAAAATATGCAGCTTTAAATTCG

Themis: F-TTCGAGCTGCCCATGAAT;

  R-TCCATTGTCAGGTATGGAGTTTT

Atp5b: F-CTATGACCATCTCCCGGAACA;

  R-CAGCTTGTCAGCCTTTGCC

### Western blot

Cells were harvested in lysis buffer containing a cocktail of protease and phosphatase inhibitors (Complete Mini, EDTA-free; Roche). Proteins were separated on 10% NuPAGE midi gels (Invitrogen) and blotted onto nitrocellulose membranes. Membranes were probed with anti-Themis rabbit polyclonal antibody that recognizes the C-terminal portion of mouse Themis (kindly provided by Paul E Love), or with a monoclonal antibody that recognizes the N-terminal portion of human Themis (Sigma) and then probed with β-actin mAb antibody (clone AC-15, Sigma), before incubation with horseradish peroxidase-conjugated secondary antibodies (Amersham). Signals were revealed with SuperSignal West Pico chemiluminescent substrate (Pierce).

### Sequencing

Sequencing of *Themis* and *Ptprk* coding regions was performed according to data available on genomic databases. Total RNA was extracted (RNeasy minikit, Qiagen), and reverse transcribed (SuperScript III first-strand synthesis kit, Invitrogen). Sequencing reactions were carried out with the BigDye Terminator version 3.1 (Applied Biosystems) and recorded on ABI 3130XL. Sequences were analyzed with Vector NTI software (InforMax).

### Proliferative responses, cytokine assays, and Treg suppression assays

For cytokine production assays, total CD4 T cells or naive CD62L^+^ CD4 T cells (10^5^/well) were purified and stimulated with plate-bound anti-TCR (R73, 1 µg/ml) and soluble anti-CD28 (JJ319, 0.01 to 0.2 µg/ml) in 96-well flat-bottom plates for 48 hours in complete RPMI (Invitrogen). Cytokine production was examined in the cell supernatants by Luminex multiplex kit (Millipore). For Treg suppression assays, CD4^+^ CD25^bright^ were sorted using a FACS Aria II-Sorp (BD Biosciences). Co-culture experiments were performed using naive Lewis CD4 T cells (10^5^/well) stained with CFSE (Peprotech) as effector cells. These cells were stimulated with irradiated syngeneic APC (0.5×10^5^/well; T cell-depleted splenocytes) and plate-bound anti-CD3 mAb (G4.18; 0.5 µg/ml) in the presence of 0.5×10^5^ Treg purified from the spleen or thymus. CFSE dilution was estimated by flow cytometry after 60 hours of culture. Proliferation was also analyzed by [3H] Thymidine incorporation, added during the last 18 hours of culture.

### Transfer of wild-type CD25^bright^ CD4^+^ T cells

Four week-old BN^m^ rats were transferred with PBS (control) or with 4.10^6^ of FACS-sorted CD25^bright^ CD4^+^ T cells purified from the spleens and LN of BN rats. 8 weeks after transfer, BN^m^ rats were sacrificed and intestinal inflammation was assessed as described below. Samples of duodenum were collected to perform microscopic analyses and dosage of cytokine concentrations.

### Histology and immuno-histochemistry

Skin, liver, pancreas, lung, heart, brain, testis, thyroid, the entire gastrointestinal tract, aorta, spleen, thymus and LN were dissected and fixed in 4% paraformaldehyde before embedding in paraffin and processing for conventional hematoxylin-eosin staining. Selected panels of serial sections were processed for immuno-histochemical labeling with anti-CD3 (polyclonal rabbit serum, IR503, Dako), B220 (OX33 hybridoma supernatant) and CD68 (ED1 hybridoma supernatant). Two pathologists evaluated the intestinal lesions independently using Wallace score.

### Detection of autoantibodies

For the detection of autoantibodies directed against gut, liver, lung or kidney, tissues isolated from SCID/Beige BALB/c mice were snap frozen in liquid nitrogen and 3–5 µm sections were cut using a cryomicrotome. Sections were fixed in acetone at −20°C for 20 min and stored at −80°C. Sera diluted in PBS 0.1% BSA were applied at the chosen dilution (1/200) for 60 min at room temperature. After rinsing twice in PBS, slides were incubated for 30 min with a goat anti-rat immunoglobulin G coupled to Alexa Fluor 488 (Jackson Immunoresearch). Slides were then washed twice in PBS before mounting in anti-fading medium and direct observation with immunofluorescence microscopy. The intensity of immunofluorescent staining was blindly evaluated by O.A.

### Quantification of myeloperoxidase activity and cytokine levels in intestinal tissues

MPO activity was measured as an index of granulocyte infiltration. Briefly, tissue samples were homogenized in a solution of 0.5% hexadecyltrimethylammonium bromide and supernatants were added to O-dianisidine dihydrocholoride solution supplemented with 1% hydrogen peroxide. Optical density readings were taken at 450 nm. For cytokine analysis, intestinal samples were homogenized with Precellys beads (Ozyme) in 250 µL of cell lysis buffer (Cell Signaling Technology) containing a cocktail of protease and phosphatase inhibitors (Complete Mini, EDTA-free; Roche). Supernatants were assessed for cytokines by Luminex multiplex kit (Millipore) and for protein levels using Bradford Reagent (Bio-Rad).

### Statistical analysis

Results were expressed as means ± s.e.m. Comparisons between groups were done by the unpaired Student's *t test* using Prism software. Fisher's exact test was used to analyze the prevalence and macroscopic scores in BN^m^ rats having received or not normal regulatory T cell from BN rats. Results were considered statistically significant when the p value was ≤0.05. ns: not significant; *: p≤0.05; **: p≤0.01; ***: p≤0.001.

## Supporting Information

Figure S1 BN^m^ rats show a lymphopenia restricted to CD4 T cells. Absolute numbers of total, CD4+ and CD8+ T cells, and B cells in submandibular lymph nodes from BN (black columns; n = 4) and BN^m^ (white columns; n = 4) rats. Data are representative of four independent experiments.
(EPS)

Figure S2 BN^m^ rat thymic epithelium is able to support CD4 SP cells differentiation. Irradiated BN or BN^m^ recipients were reconstituted i.v. with 8.10^7^ T cell-depleted bone marrow cells originating from (LEW×BN) F1 rats. Recipients were sacrificed 16 weeks after transfer. Absolute numbers of lymphocytes in spleens of lethally irradiated BN (black columns; n = 4) and BN^m^ (white columns; n = 4) rats reconstituted with (LEW×BN) F1 rats T cell-depleted bone marrow cells. Data are representative of three independent experiments.
(EPS)

Figure S3 BN^m^ rats spontaneously develop features of inflammatory bowel disease. (A) Autoantibodies against SCID/Beige BALB/c mouse colon, ileum, lung, kidney and liver tissues detected after staining with sera (dilution 1/200) from BN^m^ (top) and BN (bottom) rats and goat anti-rat immunoglobulin G coupled to Alexa Fluor 488 (ml: muscular layer, LP: Lamina propria, L: lumen, v: venule, a: arteriole, g: glomerulus). (B) Prevalence of histological lesions of the disease in BN (black squares) and BN^m^ (open circles) rats according to age. 5–7 weeks of age: 13 BN, 21 BN^m^; 8–10 weeks of age: 28 BN, 49 BN^m^; 11–12 weeks of age: 25 BN, 23 BN^m^; 34 weeks of age: 6 BN, 6 BN^m^ (C) Duodenum, jejunum, ileum and colon microscopic scores of 8–12 week-old BN (black squares, n = 55) and BN^m^ rats (open circles, n = 72).
(EPS)

Figure S4 BN^m^ rats exhibit spontaneous T cell activation and skewed cytokine production. (A) Proportions of OX40^high^, CD62L^low^, CD45RC^low^ and CD25^high^ among CD4 T cells from mLN of BN (n = 4) and BN^m^ (n = 8) rats. (B) Proliferation was assessed by [3H] Thymidine uptake in total CD4 T cells isolated from BN (n = 4) and BN^m^ (n = 4) mLN and stimulated for 48 hours with anti-TCR mAb and increasing concentrations of anti-CD28 mAb (left panel). Cytokine production by CD4 T cells stimulated with anti-TCR mAb and 0.2 µg/ml of anti-CD28 mAb for 48 hours (right panels). (C) Four-week-old BN^m^ rats were injected with OX38, an anti-CD4 depleting mAb (300 µg per 100 g of body weight), 3 times a week (grey columns, n = 10) or PBS (white, columns n = 12). The proportion of CD4 T cells in PBMC was tested by flow cytometry three weeks after the first anti-CD4 injection (left panel). IBD development tested 8 weeks after the first anti-CD4 injection by analyzing the prevalence, the length of small intestine and the macroscopic and microscopic scores of the duodenum (right panels).
(EPS)

Figure S5 The suppressive activity of BN^m^ CD4^+^ CD25^+^ Treg is severely impaired. (A) Percentages of Foxp3+ CD4 SP in thymus, spleen and mLN from BN (n = 7) and BN^m^ (n = 13) rats. Data are representative of three independent experiments. (B) Naive purified BN CD4 T cells were stimulated with irradiated syngeneic APC and coated anti-CD3 in presence of decreased numbers of FACS-sorted CD4+ CD25+ Treg and cultured in 96-well round-bottom plates for 48 hours. Proliferation was analyzed by [3H] Thymidine incorporation during the last 18 hours. The data represent the suppressive activity of CD25+ CD4+ T cells from BN (black squares) and BN^m^ rats (open circles) in co-culture experiments using BN naive CD4 T cells as effectors. Data are representative of three independent experiments.
(EPS)
